# Intraperitoneal Chemotherapy of Peritoneal Carcinomatosis Using Pressurized Aerosol as an Alternative to Liquid Solution: First Evidence for Efficacy

**DOI:** 10.1245/s10434-013-3213-1

**Published:** 2013-09-05

**Authors:** Wiebke Solass, Reinhold Kerb, Thomas Mürdter, Urs Giger-Pabst, Dirk Strumberg, Clemens Tempfer, Jürgen Zieren, Matthias Schwab, Marc André Reymond

**Affiliations:** 1 Institute of Pathology, Ruhr-University Bochum, Bochum, Germany; 2 Dr. Margarete Fischer-Bosch Institute of Clinical Pharmacology, Stuttgart, Germany; 3 Department of Clinical Pharmacology, University Hospital, Tübingen, Germany; 4 Department of Surgery, Marienhospital Herne, Ruhr-University Bochum, Bochum, Germany; 5 Department of Internal Medicine, Oncology and Hematology, Marienhospital Herne, Ruhr-University Bochum, Bochum, Germany; 6 Department of Gynaecology and Obstetrics, Marienhospital Herne, Ruhr-University Bochum, Bochum, Germany

## Abstract

**Background:**

Peritoneal carcinomatosis (PC) is an unmet medical need. Despite recent improvements, systemic chemotherapy has limited efficacy. We report the first application of intraperitoneal chemotherapy as a pressurized aerosol in human patients.

**Methods:**

Three end-stage patients with advanced PC from gastric, appendiceal, and ovarian origin were treated as a compassionate therapy. All patients had received previous systemic chemotherapy. A pressurized aerosol of CO_2_ loaded with doxorubicin 1.5 mg/m^2^ and cisplatin 7.5 mg/m^2^ (pressurized intraperitoneal aerosol chemotherapy, PIPAC) was applied into the abdomen for 30 min at a pressure of 12 mmHg and a temperature of 37 °C.

**Results:**

No side-effects >2 CTCAE were observed, and the procedures were well tolerated. Early hospital discharge was possible (days 2–5). Nuclear presence of doxorubicin was documented throughout the peritoneum, reaching high local concentration (≤4.1 μmol/g) and plasma concentration was low (4.0–6.2 ng/ml). PIPAC created no significant adhesions, could be repeated, and was applied 6×, 4×, and 2×. Two patients showed a complete and one a partial histological remission. Mean survival after the first PIPAC was 288 days. One patient is alive after 567 days.

**Conclusions:**

PIPAC shows superior pharmacological properties with high local concentration and low systemic exposure. PIPAC can induce regression of PC in chemoresistant tumors, using 10 % of a usual systemic dose.

**Electronic supplementary material:**

The online version of this article (doi:10.1245/s10434-013-3213-1) contains supplementary material, which is available to authorized users.

Life expectancy in peritoneal carcinomatosis (PC) is limited due to advanced tumor stage and poor therapeutic response. Resistance of PC to systemic chemotherapy (SC) is explained by molecular mechanisms and by limited drug distribution.^1^,^2^ This is the rationale for locoregional therapy combining cytoreductive surgery (CRS) with intraperitoneal chemotherapy (IPC).^3^ However, this approach is debated: IPC is hampered by limited drug distribution within the abdominal cavity and poor penetration into PC nodules.^4^,^5^ Thus, the benefit of combined CRS and IPC compared with CRS alone might be marginal.^6^

We have proposed to apply chemotherapy as a pressurized aerosol within the abdominal cavity to take advantage of following physical properties: applying an aerosol allows a homogeneous repartition of the substance within a closed space; generating an artificial pressure gradient counterbalances tumoral interstitial fluid pressure, an obstacle in cancer therapy.^7^,^8^ In the large animal model, pressurized aerosol improved both distribution of a vital staining within the abdominal cavity, and depth of penetration into the peritoneum, as compared to peritoneal lavage with a liquid solution.^9^ When treating human PC ex vivo, we achieved a superior distribution onto the peritoneum and a better penetration into PC nodules than IPC.^10^ This was the rationale for the first application in the human patient.

## Methods

### Patients

Pressurized intraperitoneal aerosol chemotherapy (PIPAC) was offered as a treatment option to three patients suffering from a fatal disease for which no satisfactory alternative therapy was available, pursuant to the individual compassionate use of medicinal products according to the German Medical Act (AMG) and with documented favorable opinion by the Ethics Committees of the University of Münster, Germany. Patients were evaluated by our multidisciplinary team before onset of treatment and provided written, informed consent. Clinical and histological confirmation of PC, including small bowel involvement, was required. No patient had parenchymatous metastases. Patient histories are summarized in Table 1. PIPACs were performed between November 2011 and March 2013.

**Table 1:** Patient characteristics and therapy summary

Patient	Sex	Age at first PIPAC	Diagnosis	First diagnosis	Previous surgery	Previous chemotherapy regimen	Karnovsky before therapy (%)	PCI before PIPAC therapy	PIPAC procedures (n)	Secondary CRS	Adverse effects (Grade CTAEC)	Intraperitoneal tumor remission: macroscopy	Intraperitoneal tumor remission: histology	Tumor ascites control	Max. Karnovsky after therapy (%)	Status	Cause of death	Survival (days)
1	M	38	Gastric Ca, signet ring	2009 (2 years)	Gastrectomy, LAD D2	ECF, paclitaxel (disease progression)	40	6	2	No	Fever (2), vomiting (2), Pain (2)	CR	CR	N/A	40	Dead	Metastasis, cachexia	109
2	M	45	Appendix Ca, signet ring	9.2011 (6 weeks)	Ileo-caecal resection	5-FU (adverse effects)	40	16	4	Yes	Bowel perforation (4)^a^	PR	PR	N/A	70	Dead	Bowel obstruction	187
3	F	73	Ovarian Ca	2001 (10 years)	Hysterectomy, adnexectomy, LAD	Multiple regimen	40	14	6	No	Fatigue (1)	CR	CR	Yes	90	Alive	N/A	567

*PIPAC* pressurized intraperitoneal aerosol chemotherapy, *PCI* peritoneal carcinomatosis index, *CRS* cytoreductive surgery, *N/A* no ascites, *LAD* lymphadenectomy, *PR* partial (intraperitoneal) remission, *CR* complete (intraperitoneal) remission

^a^Bowel perforation after CRS combined with PIPAC

### Surgical Procedures

**Fig. 1:**
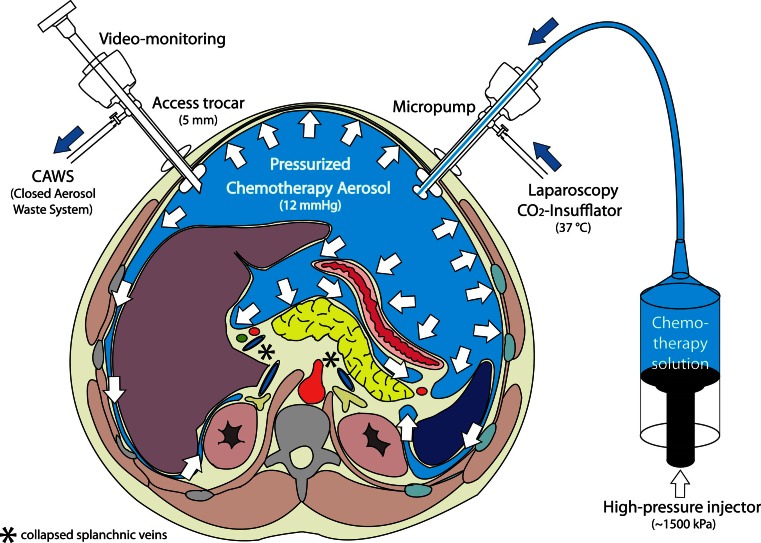
Pressurized intraperitoneal aerosol chemotherapy (PIPAC). The procedure is performed in an operating room equipped with laminar air-flow and is remote-controlled. In a first step, a normothermic capnoperitoneum is established with a pressure of 12 mmHg at body temperature. A chemotherapy solution (about 10 % of a normal systemic dose) is nebulized with a micropump into the tightly closed abdominal cavity, and maintained for 30 min. The toxic aerosol is then exhausted through a closed system and released into the external environment

PIPAC is described in Fig. 1. After insufflation of a 12 mmHg of capnoperitoneum at 37 °C, two balloon trocars (Applied Medical, Düsseldorf) were placed. Explorative laparoscopy was performed as usual and PC index was determined.^11^ Parietal biopsies were taken and ascites was removed. A nebulizer (MIP, Reger Medizintechnik, Rottweil) was connected to a high-pressure injector (Injektron 82M, MedTron, Saarbruecken) and inserted into the abdomen through a trocar. A pressurized aerosol containing cisplatin (Hexal, Barleben) at a dose of 7.5 mg/m^2^ body surface in 150 ml NaCl 0.9 % was applied immediately followed by doxorubicin (Hexal, Barleben) 1.5 mg/m^2^ in 50 ml NaCl 0.9 %. Then, the system was kept in steady-state for 30 min (application time). Toxic aerosol was exhausted over a closed system. Trocars were retracted. PIPAC was repeated two to five times at various time intervals. Occupational health safety aspects are described elsewhere.^12^

### Safety and Efficacy Assessments

Assessments for patient safety and tolerability were performed from days 1–5 after treatment and included medical examination and routine laboratory measures. Adverse effects were graded according to the NCI Criteria for Adverse Events (CTCAE).^13^ Tumor response was assessed by laparoscopy with macroscopic assessment and histology, as part of the next PIPAC cycle. Patients were followed up for analysis until June 20, 2013 or until death.

### Histology

Biopsies were analyzed for possible tumor response by conventional HE microscopy.

### Clinical Pharmacology

Analysis is based on eight PIPAC in three patients. Blood samples were drawn before, during, and up to 12 h after start of PIPAC. At the end of PIPAC, biopsies from peritoneal tissue and tumor nodules were snap-frozen. Doxorubicin plasma levels were determined by UHPLC-MS/MS using [13C2H3]-doxorubicin as internal standard. Pharmacokinetics parameters were derived by non-compartmental analysis (WinNonLin 6.3, Pharsight, Cary, NC, USA). The area under the plasma concentration-time curve (AUC) was calculated by the linear trapezoidal rule.

## Results

### Patient 1

**Fig. 2:**
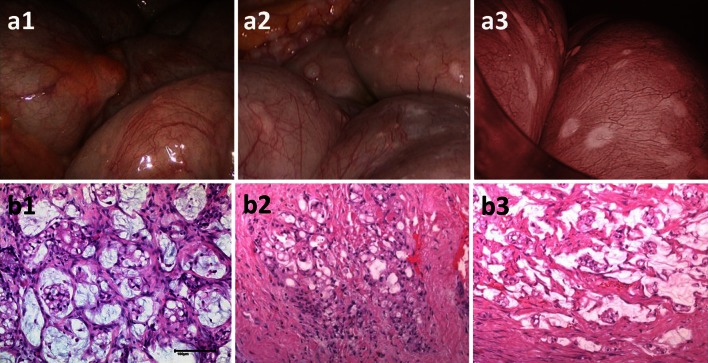
Macroscopical and histological response after PIPAC. Forty-five year male patient with diffuse peritoneal carcinomatosis (PCI = 16) from a signet-ring cells appendiceal cancer with inaugural small bowel obstruction. Macroscopy before (**a1**), after 1st (**a2**) and after 3rd PIPAC (**a3**) showing regression of small bowel PC nodules. Histology shows vital tumor before PIPAC (**b1**), inflammatory reaction with nodular sclerosis after 1st PIPAC (**b2**) and large areas of devitalized tumor after 3rd PIPAC (**b3**). *Scale bar* 100 µm

A 45-year-old male patient was operated because of acute bowel obstruction due to PC from signet-ring appendiceal cancer. Postoperative high-dose chemotherapy (5-FU) was interrupted due to acute heart failure. After recovery, Karnovsky index was 40 %. First PIPAC was performed, showing a PCI of 16 (Fig. 2a1, b1). Four weeks later, second PIPAC showed stable disease. Four weeks later, 3rd PIPAC showed hard, glassy nodules (a2), histology showed regressive changes with nodular sclerosis (b2). Six weeks later, small bowel nodules were regressive (a3), histology showed regressive changes with 60 % vital cells and large devitalized areas (b3). Complete CRS was performed, and fourth PIPAC administered. A postoperative bowel perforation required surgical revision. After recovery, the patient developed bowel obstruction and tumor progression was confirmed by laparotomy. He died 187 days after first PIPAC.

### Patient 2

A 38-year-old male patient with a 2-year history of signet-ring gastric cancer, gastrectomy, and two chemotherapy lines presented with tumor progression and end-stage disease requiring parenteral nutrition. Karnovsky index was 40 %. A PCI of 6 was documented at first PIPAC (Supplementary material 1, a1). Four weeks later, during the second PIPAC, macroscopy showed complete remission (a2), and multiple biopsies confirmed absence of tumor cells (b2). Eight weeks later, the patient developed liver and bone metastases and died 109 days after the first PIPAC. Two weeks before death, abdomen CT showed no evidence of PC.

### Patient 3

**Fig. 3:**
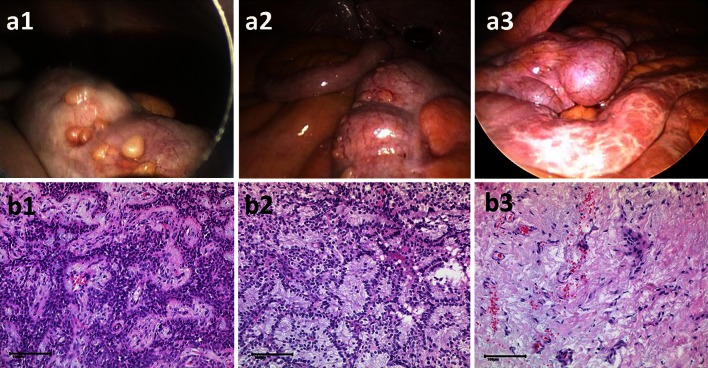
73-year-old patient (patient 3) with therapy-resistant peritoneal carcinomatosis and malignant ascites of ovarian origin. Shown is the macroscopic (*upper* fig) and histological (HE staining, *lower* fig) appearance before (*left* column), after PIPAC (*middle* column) and at later stages of follow-up (*right* column). **a**, **b** Show partial and then complete macroscopic and microscopic tumor remission after repeated PIPAC. *Scale bar* 100 µm

A 73-year-old female patient with a 10-year history of ovarian cancer, surgery, and multiple chemotherapy regimens presented with tumor progression and hemorrhagic ascites. Karnovsky index was 40 %. At first PIPAC, a PCI of 14 was documented and 2.5 l ascites removed (Fig. 3a1, b1). At second PIPAC after 4 weeks, PC nodules were hard and glassy, and ascites volume dropped to 1 l. Histology showed no regression (not shown). Six weeks later at the third PIPAC, ascites was <500 ml (a2), and histology showed partial tumor response with fibrotic reaction (b2). Karnoswky index was 90 %. Eight months after first PIPAC, fourth PIPAC showed complete remission (a3), and multiple biopsies showed apoptotic inflammatory cells (b3) but no tumor. After 15 months of follow-up, the asymptomatic patient underwent control laparoscopy; tumor was documented in two of five peritoneal biopsies, so fifth PIPAC was applied. Six weeks later, a single, 6-cm, large tumor node was resected. All other biopsies were negative; sixth PIPAC was applied. After 567 days, the patient is alive with an excellent quality of life.

### Safety

All 11 PIPAC procedures were technically easy to perform. For PIPAC alone, mean operating time was 93 ± 13 min. No intraoperative complication was noted. PIPAC alone was very well tolerated, no adverse effect >2 CTCAE was noted (Table 1). After PIPAC alone, patients were discharged from hospital 2–5 days after treatment. In two instances, PIPAC was combined with another operation (small-bowel resection, complete cytoreductive surgery): in the latter case, a postoperative bowel perforation (see Patient 1 above) required surgical revision.

### Clinical Pharmacology

**Fig. 4:**
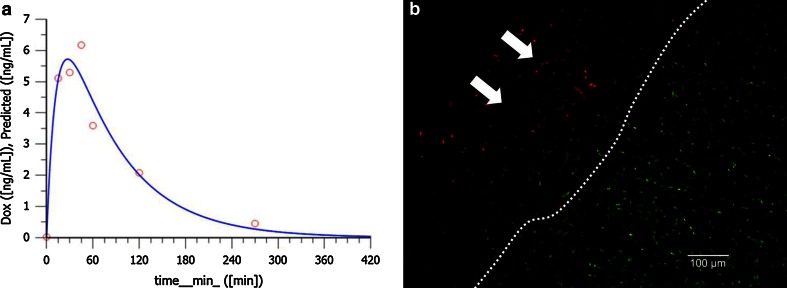
Local and systemic doxorubicin exposure during PIPAC. Local disposition is high with 1.70 ± 1.45 µg/g. In **a** fluorescence microscopy shows a nuclear presence of doxorubicin up to 500 µm depth. *Red* doxorubicin. *Green* picogreen nuclear counterstaining. *Scale bar* 100 µm. **b** Shows a typical pharmacokinetic profile in peripheral venous blood after PIPAC with doxorubicin 1.5 mg/m^2^ body surface for 30 min at an intraabdominal pressure of 12 mmHg. Peak doxorubicin plasma concentrations were low (4.0–6.2 ng/ml). *Line* predicted profile. *Dots* experimental values

The plasma concentration-time curve fitted best to a two-compartment model with first-order absorption. Peak doxorubicin plasma concentrations were low (4.0–6.2 ng/ml) and were reached with the end of nebulisation. Doxorubicin was eliminated from the body with a clearance (Cl/F) of 2.6–6.0 ml/min. Half-lives and AUCs ranged from 86 to 468 min and 415 to 915 ng/ml min, respectively (Fig. 4a). Tissue concentration of doxorubicin was high (mean 1.7 μg/g) and variable (SD ± 1.45 μg/g). In tumor nodules, fluorescence microscopy showed nuclear presence of doxorubicin up to 500 μm depth (Fig. 4b) and throughout the whole peritoneal layer into the properitoneal fatty tissue (>600 μm, Supplementary material 2). Highest concentration was achieved within 100–200 μm from the surface.

## Discussion

To our knowledge, this is the first report of successful application of chemotherapy as a pressurized aerosol within the abdomen of human patients. It has been predicted that innovative concepts overcoming pharmacologic limitations of IPC could improve, perhaps dramatically, its efficacy.^5^ A superior dose–response ratio after PIPAC could be expected from preclinical data. In the human patient, plasma concentration-time profile analysis now confirms such superior ratio between dose, systemic, and local drug concentration: PIPAC required only 1/10 of the doxorubicin dose to achieve higher tumor concentrations (0.03–4.1 μmol/g) as reported for HIPEC (0.02 μmol/g).^15^ In contrast, systemic availability of doxorubicin after PIPAC and HIPEC were equal as indicated by the approximately ten times lower maximal plasma concentration after PIPAC.

We and others have reported that increasing intraperitoneal pressure enhanced particularly the uptake of drugs into the tumor, resulting in a higher local disposition.^9^,^10^,^15^,^16^ After PIPAC, doxorubicin was not only detected in significant concentrations in PC nodules, but nuclear staining was demonstrated throughout the peritoneum, up to deeply into the retroperitoneal fatty tissue. Another explanation for superior local disposition could be the high drug concentration in the aerosol. Although used in only 1/10 of the total dose, doxorubicin concentration in the aerosol (52 μM) is three times higher as in the intraperitoneal fluid usually used in HIPEC (18 μM) without impairing tolerability, which was reported after applying higher concentrations of IPC.^15^,^17^

Tumor response was observed in all three cases after PIPAC, as a consequence of the well-documented antitumor activity of doxorubicin and cisplatin and the superior local disposition. However, we were surprised by the extent of macroscopic and microscopic response in these multidrug-resistant tumors. We documented a complete remission of PC in two patients, which was indeed unexpected.

At this stage, it would be clearly premature to claim that combined PIPAC with cisplatin and doxorubicin improves survival in advanced PC. However, in our three patients with multiresistant tumors, low performance index, and very limited life expectancy, we observe a mean survival of more than 288 days. Remarkably, 567 days after her first PIPAC patient 3 is still alive.

In sharp contrast to HIPEC, PIPAC was very well tolerated and the only severe adverse effect observed was a bowel perforation after CRS (Table 1). Otherwise, postoperative courses were uneventful, with early hospital discharge.

PIPAC might create synergies with SC. Liver and renal tests showed neither acute nor cumulative toxicity after PIPAC, which appears reasonable bearing in mind the 90 % dose reduction compared with conventional SC.^19^ Moreover, PIPAC permits repeated cycles of IPC and therefore might allow effective regimen combining SC and PIPAC. Importantly, repeated laparoscopy enables objective staging, assessment of therapeutic response, and adaptation of further therapy accordingly, which was barely possible until now. Finally, considering that all three patients were in poor physical condition with a low performance index, PIPAC might allow therapy in polymorbid patients—when SC is contraindicated.

We observed tumor regression even in platin-resistant tumors, after application of cisplatin and doxorubicin. This is not surprising since drug effect is usually dose-dependent. PIPAC might become an alternative therapy for platin-resistant tumors, in particular in women with ovarian cancer where tumor progression is diagnosed after first-line therapy with carboplatin–Taxol. Repeated intraoperative analysis of the environmental air showed that PIPAC is safe for staff and meets the requirements of the German working safety regulations.^12^

## Conclusions

These early data are promising: PIPAC can induce remission in end-stage, therapy-resistant PC, and first safety data are encouraging. PIPAC is well tolerated, a decisive feature in patients with limited life expectancy. By requiring only 10 % of the dose of conventional IPC, PIPAC shows an excellent local distribution with low systemic exposure. Furthermore, PIPAC permits repeated cycles of IPC as well as objective tumor staging and response assessment. PIPAC is easy to use. PIPAC is complying with EC occupational safety regulations. The potential of this generic technology for a variety of indications and drugs has now to be determined with adequate studies.

## Electronic supplementary material

Below is the link to the electronic supplementary material.

Supplementary Fig. 1 A 38-year male patient (patient 1) with a 2 year history of signet-ring gastric cancer, gastrectomy and two chemotherapy lines. Macroscopy before **a1** and after 1st PIPAC **a2** showing vanishing of small bowel PC nodules. Histology **b** confirms complete remission of PC 4 weeks after PIPAC. *Scale bar* 100 µm (JPEG 98 kb)

Supplementary Fig. 2 Fluorescence microscopy shows doxorubicin nuclear staining with doxorubicin (*red*) throughout the whole peritoneal layer into the properitoneal fatty tissue (>600 µm). *Green* picogreen nuclear counterstaining. *Scale bar* 100 µm (TIFF 313 kb)
