# Supplementary figures and images for: Intraperitoneal Chemotherapy of Peritoneal Carcinomatosis Using Pressurized Aerosol as an Alternative to Liquid Solution: First Evidence for Efficacy
Source: Ann Surg Oncol. 2013 Sep 5;21(2):553–9. doi: 10.1245/s10434-013-3213-1 (PMC3929768; doi:10.1245/s10434-013-3213-1)

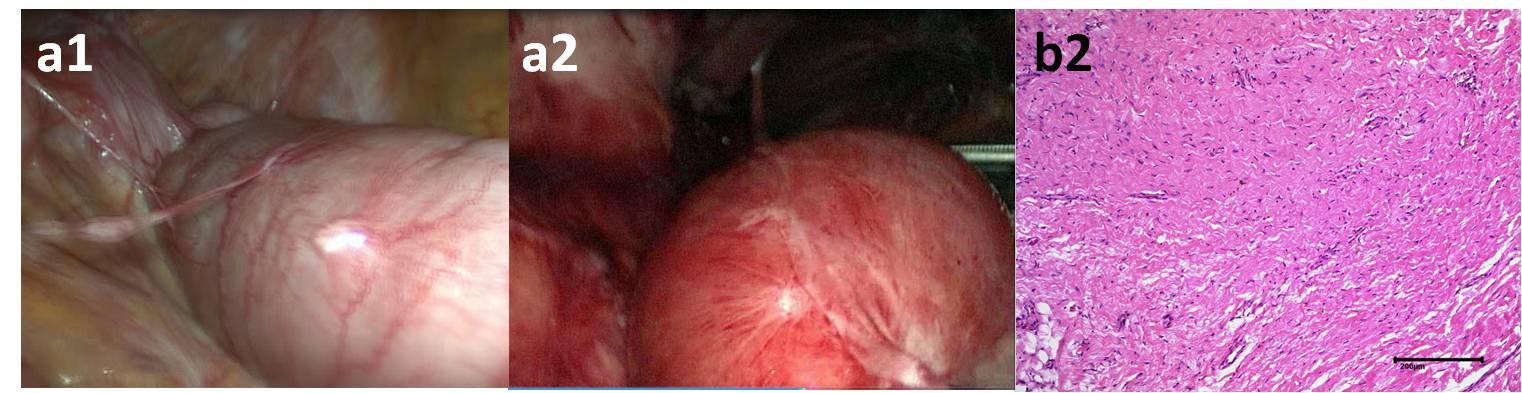

Supplement: Supplementary file 1 — A 38-year male patient (patient 1) with a 2 year history of signet-ring gastric cancer, gastrectomy and two chemotherapy lines. Macroscopy before a1 and after 1st PIPAC a2 showing vanishing of small bowel PC nodules. Histology b confirms complete remission of PC 4 weeks after PIPAC. Scale bar 100 µm (JPEG 98 kb) [file 10434_2013_3213_MOESM1_ESM.jpg]

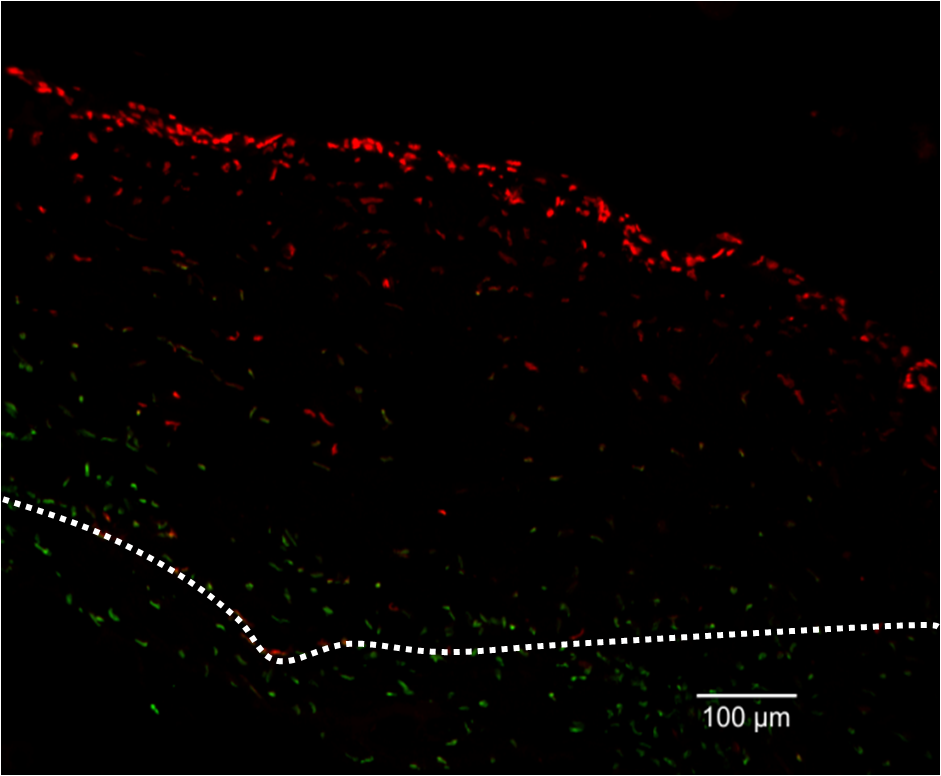

Supplement: Supplementary file 2 — Fluorescence microscopy shows doxorubicin nuclear staining with doxorubicin (red) throughout the whole peritoneal layer into the properitoneal fatty tissue (>600 µm). Green picogreen nuclear counterstaining. Scale bar 100 µm (TIFF 313 kb) [file 10434_2013_3213_MOESM2_ESM.tif]
